# Quantum Machine Learning—Quo Vadis?

**DOI:** 10.3390/e26110905

**Published:** 2024-10-24

**Authors:** Andreas Wichert

**Affiliations:** Department of Computer Science and Engineering, INESC-ID & Instituto Superior Técnico, University of Lisbon, 2744-016 Porto Salvo, Portugal; andreas.wichert@tecnico.ulisboa.pt

**Keywords:** quantum machine learning, basis encoding, amplitude encoding, input destruction problem, HHL, quantum kernels, variational algorithm

## Abstract

The book *Quantum Machine Learning: What Quantum Computing Means to Data Mining*, by Peter Wittek, made quantum machine learning popular to a wider audience. The promise of quantum machine learning for big data is that it will lead to new applications due to the exponential speed-up and the possibility of compressed data representation. However, can we really apply quantum machine learning for real-world applications? What are the advantages of quantum machine learning algorithms in addition to some proposed artificial problems? Is the promised exponential or quadratic speed-up realistic, assuming that real quantum computers exist? Quantum machine learning is based on statistical machine learning. We cannot port the classical algorithms directly into quantum algorithms due to quantum physical constraints, like the input–output problem or the normalized representation of vectors. Theoretical speed-ups of quantum machine learning are usually analyzed in the literature by ignoring the input destruction problem, which is the main bottleneck for data encoding. The dilemma results from the following question: should we ignore or marginalize those constraints or not?

## 1. Introduction

Deep learning has achieved tremendous successes; it is based on error minimizing algorithms that approximate a distribution of a population. The approximation is based on the error minimization of the prediction of a very large training sample, with the assumption that the large sample describes the population sufficiently well. The error minimization is based on a loss function and the back-propagation algorithm. The back-propagation algorithm applied to deep learning architectures requires huge computational resources in hardware, energy, and time. The promise of quantum machine learning is that it will overcome these problems due to quadratic or even exponential speed-up in time and the possibility of compressed data representation. However, can we really apply quantum machine learning for real-world applications? What are the algorithmic constraints of quantum machine learning? Currently there is a huge body of literature on quantum machine learning, which we are unable to review. Instead we will deal with four fundamental categories of quantum machine learning including quantum encoding.

- The process of transferring classical data into quantum states is an essential step in quantum algorithms. This process is called quantum encoding or quantum state preparation. Binary patterns representation of a training sample using quantum encoding and the application of the Grover’s algorithm is presented. It leads to quadratic speed-up of popular machine learning algorithms, like k-nearest neighbor, clustering, and associative memory.
- We describe the representation of a training sample by amplitude encoding. It is used in the quantum algorithm for linear systems of equations (HHL) based on Kitaev’s phase estimation algorithm leading to an exponential speed-up. Since almost all machine learning algorithms use some form of a linear system of equations, it is assumed that the HHL algorithm is going to be one of the most useful subroutines.
- Quantum kernels that are not based on the kernel trick but map the vectors directly into high-dimensional space and may lead to new kernel functions.
- Variational approaches are characterized using a classical optimization algorithm to iteratively update a parameterized quantum trial solution that can open new insights in real-world problems, like the study of the behavior of complex physical systems that cannot be tackled with classical machine learning algorithms.

## 2. Binary Patterns and the Grover’s Algorithm

Basis encoding encodes a *n* dimensional binary vector to a *n*-qubit quantum basis state. Ventura and Martinez [1,2] proposed a method to encode *m* binary linear independent vectors with dimension *n* (with n>m) into a superposition of a *n*-qubit quantum state. We describe the simplified method by [3]. The procedure successively divides a present superposition into processing and memory branches. The input patterns are loaded into new generated memory branches step by step. The cost of the method is linear in the number of stored patterns and their dimension [4]. At the initial step, the system is in the basis state with load qubits, memory qubits and the control qubits $c_{1},c_{2}$

$$|memory;c_{2},c_{1};load\rangle$$

(using little endian notation). The basis states are split step by step using the control qubits $c_{1},c_{2}$ until the required superposition is present

$$\frac{1}{\sqrt{m}}\sum_{j=1}^{m}{|memory;c_{2},c_{1};load\rangle }_{j}$$

and with the memory register in the required superposition

$$\frac{1}{\sqrt{m}}\sum_{j=1}^{m}{|memory;0,0;0\cdots 0\rangle }_{j}=\left(\frac{1}{\sqrt{m}}\sum_{j=1}^{m}{|memory\rangle }_{j}\right)⊗|0,0;0\cdots 0\rangle .$$

The processing branch is indicated by the control qubit $c_{2}$ with the value one ($c_{2}=1$) and the memory branch representing the current superposition with the control qubit $c_{2}$ with the value zero ($c_{2}=0$). The the qubit $c_{2}$ is split by the operator $CS_{p}$ represented by the parametrized U gate U(θ,ϕ,λ)= with ϕ=π,λ=π, and $\theta =\arcsin\left(\frac{1}{\sqrt{p}}\right)\cdot 2$

$$CS_{p}=CU\left(\arcsin\left(\frac{1}{\sqrt{p}}\right)\cdot 2,\pi ,\pi \right)=\left(\begin{array}{cccc} 1 & 0 & 0 & 0\\ 0 & 1 & 0 & 0\\ 0 & 0 & \sqrt{\frac{p-1}{p}} & \frac{1}{\sqrt{p}}\\ 0 & 0 & \frac{-1}{\sqrt{p}} & \sqrt{\frac{p-1}{p}} \end{array}\right)$$

with $CS_{p}|c_{2},c_{1}\rangle$

$$CS_{p}|01\rangle =|01\rangle ,\;\;\;\;CS_{p}|11\rangle =\frac{1}{\sqrt{p}}\cdot |10\rangle +\sqrt{\frac{p-1}{p}}\cdot |11\rangle .$$

**Figure 1:**
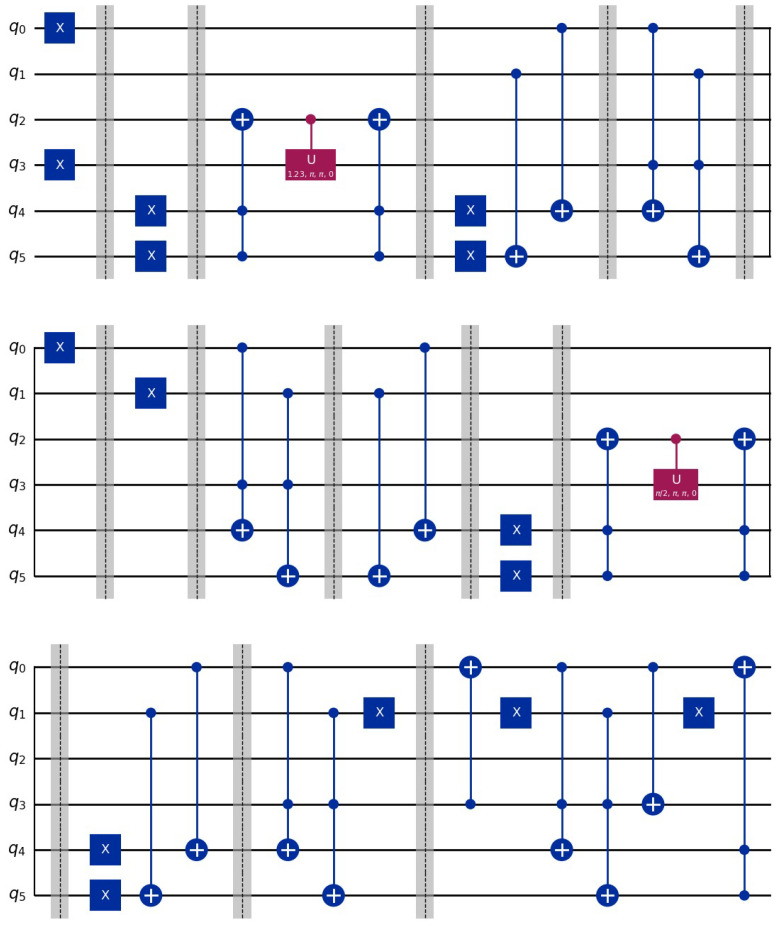
In this example, we store three binary patterns, |01〉,|10〉,|11〉. After applying the circuit the resulting state is $\frac{1}{\sqrt{3}}|0,0;1,0;0,0\rangle +\frac{1}{\sqrt{3}}|0,1;0,0;0,0\rangle +\frac{1}{\sqrt{3}}|1,0;0,0;0,0\rangle$. For more details see [5]. The dashed lines are visual indicators of the grouping of a circuit sections.

The control qubit $c_{1}$ indicates the split of the qubit $c_{2}$. Since the control qubit $c_{1}\;=\;1$ is entangled with the memory register we create the memory branch ($c_{2}=0$) with $\frac{1}{\sqrt{p}}\cdot |memory;01\rangle$ and processing branch ($c_{2}=1$) $\sqrt{\frac{p-1}{p}}\cdot |memory;11\rangle$ by the split operation on the preceding processing branch. A new pattern is stored in the generated memory register of the new generated memory branch. We repeat the procedure until we arrive in the final state

$$|\psi \rangle =\frac{1}{\sqrt{m}}\sum_{j=1}^{m}{|memory;0,0;0\cdots 0\rangle }_{j}$$

representing the binary patterns in superposition of *m* basis states. In Figure 1 we indicate a circuit for the preparation of the three states |01〉,|10〉,|11〉.

We can apply Grover’s algorithm with a quadratic speed-up of $\sqrt{m}$, since the represented *m* basis states are known to us. The constructed Grover operator amplifies only the *m* basis states and the n−m states with the amplitudes zero are unchanged.

Alternatively, we could entangle the index qubits that are in the superposition with the binary patterns.

$$|pattern_{m}\rangle ,|pattern_{m-1}\rangle ,\cdots |pattern_{1}\rangle .$$

To store *m* binary patterns we entangle the index qubits using multi-controlled NOT gates by generating $v=log_{2}\left(m\right)$ index qubits using *v* Hadamard gates

$$H^{{⊗}^{v}}{|0\rangle }^{{⊗}^{v}}=\frac{1}{\sqrt{m}}\sum_{j=1}^{m}|index_{j}\rangle .$$

and entangle *m* binary pattern with the index qubits with a resulting superposition

(1) $$\frac{1}{\sqrt{m}}\left(\sum_{j=1}^{m}|index_{j}\rangle |pattern_{j}\rangle \right).$$

Using an oracle o() we can mark the corresponding index state and un-compute the entangled patterns resulting in a state

(2) $$|\psi \rangle =\frac{1}{\sqrt{m}}\left(\sum_{j=1}^{m}{\left(-1\right)}^{o(index_{j})}\cdot |index_{j}\rangle |0\cdots 0\rangle \right).$$

We can now apply Grover’s algorithm with a quadratic speed-up of $\sqrt{m}$ to the index states that point to our binary patterns.

### 2.1. Input Destruction Problem

The naïve assumption that the speed-up is quadratic is not realistic due to the input destruction problem (ID problem) [6,7,8,9]:

- The input (reading) problem: The quantum state is initialized by state preparation of *m* binary patterns. Although the existing quantum algorithm requires only $\sqrt{m}$ steps and is faster than the classical algorithms, *m* data points must be read—prepared. Hence, the complexity of the algorithm does not decrease.
- The destruction problem: We are required to read *m* data points and are allowed to query only once because of the collapse during the measurement (destruction).

Additionally, the output quantum state in many quantum machine learning algorithms must be fully read, but measuring the state collapses it to a single value, requiring many measurements to interpret the full state.

### 2.2. Quantum Random Access Memory

To avoid input destruction problem [10] quantum random access memory (qRAM) [11] was proposed. A qRAM access basis states by copy operation [11]. A register |i〉 is queried and the *i*th binary patter is loaded into the second register

(3) $$|i\rangle |0\rangle \to \left|i\rangle \right|x^{i}\rangle ,$$

with $|x^{i}\rangle$ being a basis state representing a binary vector. The query operation can be executed in parallel with

(4) $$\frac{1}{\sqrt{m}}\sum_{i=1}^{m}|i\rangle |0\rangle \to \frac{1}{\sqrt{m}}\sum_{i=1}^{m}\left|i\rangle \right|x^{i}\rangle ,$$

with the time complexity ignoring the preparation cost of (due to the input problem) is O(log(m)). The qRAM suffers from the input destruction problem. Its usage leads to a circular argument.

## 3. Amplitude Encoding and the HHL Algorithm

Amplitude encoding encodes data into the amplitudes $\omega_{i}$ of a quantum state

(5) $$|\psi \rangle =\sum_{i=1}^{N}\omega_{i}\cdot |x\rangle .$$

For example, a complex normalized vector x

$$x=\left(\begin{array}{c} \sqrt{0.03}\\ \sqrt{0.07}\\ \sqrt{0.15}\\ \sqrt{0.05}\\ \sqrt{0.1}\\ \sqrt{0.3}\\ \sqrt{0.2}\\ \sqrt{0.1} \end{array}\right).$$

is represented as

$$|\psi \rangle =\sqrt{0.03}\cdot |000\rangle +\sqrt{0.07}\cdot |001\rangle +\sqrt{0.15}\cdot |010\rangle +\sqrt{0.05}\cdot |011\rangle +$$

$$+\sqrt{0.1}\cdot |100\rangle +\sqrt{0.3}\cdot |101\rangle +\sqrt{0.2}\cdot |110\rangle +\sqrt{0.1}\cdot |111\rangle$$

by a top-down divide strategy using parametrized rotation gates with a linear complexity. Amplitude coding can only represent normalized vectors and also suffers from the input destruction problem.

### 3.1. Quantum Random Access Memory for Amplitude Coding

An operation that would produce a copy of an arbitrary quantum state such as |ψ〉 is not possible; we cannot copy non-basis states because of the linearity of quantum mechanics. However, we can, to some extent, simulate the copy of non-basis states using quantum random access memories (qRAM) as proposed by [9]. The resulting complexity would be O(nlog(m)) where *n* is the dimension of the resulting superposition vector [9]. We divide the binary vector of the dimension $2^{m}$ into *v* substrings; each substring $code_{i}$ represents a real number by fractional representation (binary representation of a real number)

(6) $$|code_{1}code_{2}\cdots code_{n}\rangle \frac{1}{\sqrt{n}}\sum_{i=1}^{n}|i\rangle .$$

with $n=2^{m}/v$ and a fractional real number $\alpha_{i}$ that is smaller than one

$$code_{i}=\alpha_{i}<1.$$

We add an auxiliary state $|0^{⊗n}\rangle$

(7) $$|code_{1}\cdots code_{n}\rangle \frac{1}{\sqrt{n}}\sum_{i=1}^{n}|i\rangle \to |code_{1}\cdots code_{n}\rangle \frac{1}{\sqrt{n}}\sum_{i=1}^{n}\left|i\rangle \right|0^{⊗n}\rangle$$

For each $\alpha_{i}$ we preform a controlled rotation $R(\alpha_{i})$

$$\left(C\cdot \alpha_{i}|1\rangle +\sqrt{1-C^{2}\cdot \alpha_{i}^{2}}|0\rangle \right)$$

and measure. By measuring the corresponding auxiliary register with the result |1〉 we know that the resulting state is correct. However, if we measure in auxiliary register |0〉, we have to repeat the whole procedure. The success rate of measuring *n*|1〉 is very low ($0.5^{n}$) and converges to zero for large *n*. For large *n* it is simply not feasible. If we succeed, the resulting state would be

(8) $$\sum_{i=1}^{n}\alpha_{i}|i\rangle .$$

The described routine is non reversible since it is based on measurement. If the probability of success was not very low, the complexity reading *m* vectors of dimension *n* would be O(nlog(m)) ignoring the preparation costs, compared to O(n·m) on a classical RAM. However, the qRAM for amplitude coding is not feasible and does not solve the input destruction problem.

### 3.2. Quantum Algorithm for Linear Systems of Equations

Systems of linear equations can be solved by Gauss elimination with $O(n^{3})$. The approximate solution for a sparse matrix via conjugate gradient descent requires much lower costs, $\tilde{O}\left(n\right)$ [12].

By ignoring certain constraints, the quantum algorithm for linear systems of equations on a quantum computer is exponentially faster for sparse matrices than any algorithm that solves linear systems on the classical computer [10]. It is based on amplitude coding and is called the HHL algorithm according to its inventors Aram Harrow, Avinatan Hassidim, and Seth Lloyd. For an invertible complex matrix n×n *A* and a complex vector b

(9) A·x=b

we want to find x. The following constraints are present.

- The vectors |b〉 and |x〉 represented by $\log_{2}n$ qubits have a length of one in the $l_{2}$ norm with
  (10) $$|b\rangle =\frac{\sum_{i=1}^{n}b_{i}|i\rangle }{∥\sum_{i=1}^{n}b_{i}|i\rangle ∥}$$
  and
  (11) $$|x\rangle =\frac{\sum_{i=1}^{n}x_{i}|i\rangle }{∥\sum_{i=1}^{n}x_{i}|i\rangle ∥}.$$
- |b〉 has to be prepared efficiently with the cost no bigger than log(n).
- The matrix *A* is sparse.
- For the output we are interested in the global properties of |x〉 rather than the coefficients $x_{i}$.

If *A* is Hermitian $A^{*}=A$ (for real matrix $A^{T}=A$) then *A* can be represented by the spectral decomposition as

(12) $$A=\lambda_{1}\cdot |u_{1}\rangle \langle u_{1}|+\lambda_{2}\cdot |u_{2}\rangle \langle u_{2}|+\cdots +\lambda_{n}\cdot |u_{n}\rangle \langle u_{n}|.$$

and

(13) $$A^{-1}=\frac{1}{\lambda_{1}}\cdot |u_{1}\rangle \langle u_{1}|+\frac{1}{\lambda_{2}}\cdot |u_{2}\rangle \langle u_{2}|+\cdots +\frac{1}{\lambda_{n}}\cdot |u_{n}\rangle \langle u_{n}|.$$

It follows

(14) $$A^{-1}\cdot |u_{j}\rangle =\frac{1}{\lambda_{j}}|u_{j}\rangle$$

and writing |b〉 as a linear combination of the eigenvectors of *A*

(15) $$|b\rangle =\sum_{j}|u_{j}\rangle \langle u_{j}|b\rangle$$

leads to

(16) $$A\cdot |b\rangle =\sum_{j}\lambda_{j}|u_{j}\rangle \langle u_{j}|b\rangle$$

and

(17) $$|x\rangle =A^{-1}\cdot |b\rangle =\sum_{j}\lambda_{j}^{-1}|u_{j}\rangle \langle u_{j}|b\rangle .$$

We estimate the eigenvalues using Kitaev’s phase estimation algorithm. We then estimate the unknown eigenvalue $e^{2\cdot \pi \cdot i\cdot \theta_{j}}$. If we apply *U* to $|u_{j}\rangle$ we obtain

(18) $$U\cdot |u\rangle =e^{2\cdot \pi \cdot i\cdot \theta_{j}}\cdot |u_{j}\rangle =e^{i\cdot \lambda_{j}}\cdot |u_{j}\rangle .$$

This representation is similar to the evolutionary operator $U_{t}=e^{-i\cdot t\cdot H}$ for t=1 and H:=A is the Hamiltonian operator. The process of implementing a given Hamiltonian evolution on a quantum computer is called Hamiltonian simulation [13]. The challenge in Hamiltonian simulation is due to the fact that the application of matrix exponentials is computationally expensive [4]. The dimension of the Hilbert space grows exponentially with the number of qubits, and thus any operator will be of exponential dimension. The computation of the matrix exponential is difficult, and this is still a topic of considerable current research. Only for a sparse hermitian matrix *H*, the Hamiltonian simulation can be implemented efficiently. We do not need to know the eigenvector $|u_{j}\rangle$ of *U*. Since a quantum state |b〉 can be decomposed into an orthogonal basis

(19) $$|b\rangle =\sum_{j}|u_{j}\rangle \langle u_{j}|b\rangle =\sum_{j}\beta_{j}|u_{j}\rangle .$$

After applying Kitaev’s phase estimation algorithm to *U* that we estimated by the Hamiltonian simulation for each value *j* the values ${\tilde{\lambda }}_{k|j}$ approximate the true value $\lambda_{j}$. For simplicity, we assume

(20) $$\sum_{j=1}^{n}\beta_{j}\sum_{k=0}^{T-1}\alpha_{k|j}\cdot |{\tilde{\lambda }}_{k|j}|u_{j}\rangle \approx \sum_{j=1}^{n}\;\beta_{j}\cdot |{\tilde{\lambda }}_{j}\rangle |u_{j}\rangle .$$

We have to measure the corresponding eigenvalues, so that we would be able to define a circuit that performs the conditioned rotation. To conduct the conditional rotation we add an auxiliary state |0〉

(21) $$\sum_{j=1}^{n}\beta_{j}\cdot |{\tilde{\lambda }}_{j}\rangle |u_{j}\rangle |0\rangle$$

and perform the conditioned rotation on the auxiliary state |0〉 by the operator *R*

(22) $$R=\left(\begin{array}{cc} \cos\alpha & -\sin\alpha \\ \sin\alpha & \cos\alpha \end{array}\right).$$

with the relation

(23) $$\alpha =\arccos\left(\frac{C}{\tilde{\lambda }}\right)$$

with *C* being a constant of normalization. Each eigenvalue indicates a special rotation

$$\sum_{j=1}^{n}\left(\beta_{j}\cdot |{\tilde{\lambda }}_{j}\rangle |u_{j}\rangle \left(R\left({\tilde{\lambda }}_{j}^{-1}\right)\left|0\rangle \right)\right)\right)=$$

(24) $$\sum_{j=1}^{n}\left(\beta_{j}\cdot |{\tilde{\lambda }}_{j}\rangle |u_{j}\rangle \left(\frac{C}{{\tilde{\lambda }}_{j}}|1\rangle +\sqrt{1-\frac{C^{2}}{{\tilde{\lambda }}_{j}^{2}}}|0\rangle \right)\right).$$

We un-compute the phase estimation procedure, resulting in the state

$$|0\rangle \sum_{j=1}^{n}\left(\beta_{j}\cdot |u_{j}\rangle \left(\frac{C}{{\tilde{\lambda }}_{j}}|1\rangle +\sqrt{1-\frac{C^{2}}{{\tilde{\lambda }}_{j}^{2}}}|0\rangle \right)\right)=$$

(25) $$|0\rangle \sum_{j=1}^{n}\left(C\cdot \frac{\beta_{j}}{{\tilde{\lambda }}_{j}}|u_{j}\rangle |1\rangle +\sqrt{1-\frac{C^{2}}{{\tilde{\lambda }}_{j}^{2}}}\cdot \beta_{j}|u_{j}\rangle |0\rangle \right).$$

By measuring the auxiliary qubit with the result 0 we obtain

(26) $$|0\rangle \sum_{j=1}^{n}\left(\sqrt{1-\frac{C^{2}}{{\tilde{\lambda }}_{j}^{2}}}\cdot \beta_{j}|u_{j}\rangle \right)$$

and with the result 1

(27) $$C\cdot |0\rangle \sum_{j=1}^{n}\left(\frac{\beta_{j}}{{\tilde{\lambda }}_{j}}|u_{j}\rangle \right)=C\cdot |0\rangle A^{-1}|b\rangle \approx C\cdot |0\rangle |x\rangle .$$

We have to select the outcome of the measurement 1

(28) $$|x\rangle =A^{-1}|b\rangle =\sum_{j=1}^{n}\frac{\beta_{j}}{{\tilde{\lambda }}_{j}}|u_{j}\rangle$$

which requires several measurements. After the preceding measurements we cannot obtain the solution |x〉 efficiently. Obtaining the required coefficient $x_{i}$ from |x〉 would require at least *n* measurements, so the complexity of the algorithm would be O(n) which is the same cost for an approximate solution for a sparse matrix via conjugate gradient descent on a classical computer.

For example,

the solution to the problem

$$A=\left(\begin{array}{cc} 1 & -\frac{1}{3}\\ -\frac{1}{3} & 1 \end{array}\right),\;\;\;\;b=\left(\begin{array}{c} 1\\ 0 \end{array}\right).$$

is represented by

$$A^{-1}=\left(\begin{array}{cc} \frac{9}{8} & \frac{3}{8}\\ \frac{3}{8} & \frac{9}{8} \end{array}\right),\;\;\;\;x=\left(\begin{array}{c} \frac{9}{8}\\ \frac{3}{8} \end{array}\right).$$

with the normalized vector being

$$x_{n}=\frac{x}{∥x∥}=\left(\begin{array}{c} 0.948683\\ 0.316228 \end{array}\right).$$

We represent

$$|b\rangle =|0\rangle =b=\left(\begin{array}{c} 1\\ 0 \end{array}\right)$$

with

$$|u_{1}\rangle =\frac{|0\rangle -|1\rangle }{\sqrt{2}},\;\;\;\;|u_{2}\rangle =\frac{|0\rangle +|1\rangle }{\sqrt{2}}$$

and with

$$|b\rangle =\sum_{j}\beta_{j}|u_{j}\rangle =|0\rangle =\frac{1}{\sqrt{2}}\cdot |u_{1}\rangle +\frac{1}{\sqrt{2}}\cdot |u_{2}\rangle$$

$$|b\rangle =\frac{1}{\sqrt{2}}\cdot \frac{|0\rangle -|1\rangle }{\sqrt{2}}+\frac{1}{\sqrt{2}}\cdot \frac{|0\rangle +|1\rangle }{\sqrt{2}}=|0\rangle .$$

We perform conditioned rotation on the auxiliary state |0〉 by

$$R_{Y}\left(\alpha \right)=\left(\begin{array}{cc} \cos\left(\frac{\alpha }{2}\right) & -\sin\left(\frac{\alpha }{2}\right)\\ \sin\left(\frac{\alpha }{2}\right) & \cos\left(\frac{\alpha }{2}\right) \end{array}\right)$$

with measured two control qubits being |10〉 representing ${\tilde{\lambda }}_{1}=2$ and |01〉 representing ${\tilde{\lambda }}_{2}=1$,

(29) $$\alpha_{1}=2\cdot \arccos\left(\frac{1}{{\tilde{\lambda }}_{1}}\right)=2\cdot \arccos\left(\frac{1}{2}\right)=\frac{\pi }{3}$$

(30) $$\alpha_{2}=2\cdot \arccos\left(\frac{1}{{\tilde{\lambda }}_{2}}\right))=2\cdot \arccos\left(\frac{1}{1}\right)=\pi$$

The measured estimated probability value (requiring several measurements) of the HHL simulation are 0.562 and 0.0622 resulting in

$$x_{m}^{2}=\left(\begin{array}{c} \frac{0.56}{0.562+0.0622}\\ \frac{0.0622}{0.562+0.062} \end{array}\right)$$

with

$$x_{m}=\left(\begin{array}{c} 0.948869\\ 0.31567 \end{array}\right)\approx x_{n}=\left(\begin{array}{c} 0.948683\\ 0.316228 \end{array}\right).$$

## 4. Quantum Kernels

To avoid the input destruction problem quantum kernels were proposed. A quantum computer can estimate a quantum kernel and the estimate can be used by a kernel method on a classical computer, [4]. Using a quantum computer results in an exponential advantage in evaluating inner products, allowing us to estimate the quantum kernel directly in the higher dimensional space by a function ϕ(x) with

(31) $$k\left(x,y\right)={\left|\langle \phi \left(x\right)|\phi \left(y\right)\rangle \right|}^{2}.$$

For large high-dimensional space, such a procedure is not tractable on a classic computer [14]. However, classically, we do not compute the function ϕ(x); instead we compute inner products specified by the kernel k(x,y)

(32) $$k\left(x,y\right)=\Phi^{T}\left(x\right)\Phi \left(y\right)=\langle \Phi \left(x\right)|\Phi \left(y\right)\rangle .$$

and the feature space could be of infinite dimensionality

(33) $$k\left(x,y\right)=\langle \Phi \left(x\right)|\Phi \left(y\right)\rangle =\sum_{j=1}^{\infty }\phi \left(x_{i}\right)\cdot \phi \left(x\right).$$

For quantum kernels the dimension of the feature space is finite, since we map the vectors directly with the aid of a quantum computer and do not specify the kernel function. The classical data are encoded into quantum data by quantum feature maps via a parametrized quantum circuit [15]. The feature vector defines by *m* parameters of the parametrized quantum circuit $U_{\phi (x)}$

(34) $$|\phi \left(x\right)\rangle =U_{\phi (x)}{|0\rangle }^{⊗m}$$

with the dimension of ϕ(x) being $2^{m}$. If we map for an input state ${|0\rangle }^{⊗m}$ with a parametrized quantum circuit $U_{\phi (x)}$ with parameters that are defined by x and un-compute it by $U_{\phi (x)}^{†}$, the inverse of the parametrized quantum circuit $U_{\phi (x)}$, then the probability of measuring the state ${|0\rangle }^{⊗m}$ is one. If we parametrize the quantum circuit *U* by x ( $U_{\phi (x)}$) and the inverse of the parametrized quantum $U^{†}$ by y ($U_{\phi (y)}^{†}$ ) and if x and y are similar, the probability of measuring ${|0\rangle }^{⊗m}$ for the input ${|0\rangle }^{⊗m}$

(35) $$U_{\phi (y)}^{†}U_{\phi (x)}|0^{⊗m}\rangle$$

should be near 1. If x and y differ a lot, this probability is smaller. The quantum kernel is represented after measurement as

(36) $$k\left(x,y\right)={\left|\langle \phi \left(x\right)|\phi \left(y\right)\rangle \right|}^{2}=|\langle 0^{⊗m}|U_{\phi (y)}^{†}|U_{\phi (x)}|0^{⊗m}{\rangle |}^{2}$$

**Figure 2:**
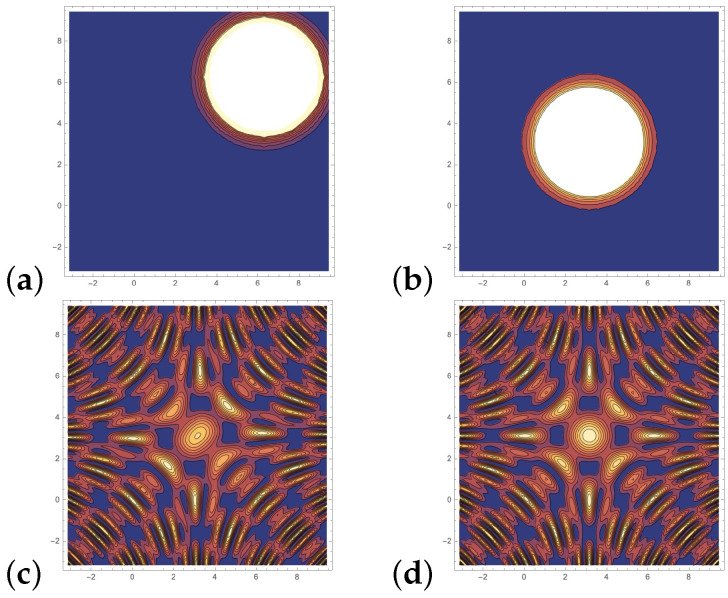
(**a**) RBF kernel with the center $y={\left(2\cdot \pi ,2\cdot \pi \right)}^{T}$. (**b**) RBF kernel with the center $y={\left(\pi ,\pi \right)}^{T}$. (**c**) Quantum kernel with the center $y={\left(2\cdot \pi ,2\cdot \pi \right)}^{T}$. (**d**) Quantum kernel with the center $y={\left(\pi ,\pi \right)}^{T}$. For the RBF kernel the position in the space defined its center; this is not the case with the quantum kernel, the center of which remains fixed; instead its wave distribution changes. In the contour plot the third dimension is indicated by the color, high values are indicated by white to yellow color, low values by blue color.

We have to measure the final state several times and record the number of $|0^{⊗m}\rangle$ and estimate the value k(x,y). The parametrized quantum circuit is based on superposition and entanglement and rotation gates that map the feature vectors in a periodic feature space resulting in periodic receptive fields with a fixed center. Quantum kernels can achieve lower training error on real data. However, this improvement leads to poor generalization on the test set and classical models can outperform quantum models [16] due to the periodic receptive fields with a fixed center, see Figure 2.

Because of this it is doubtful if quantum kennels offer any advantage over classical kernels of real-world applications.

## 5. Variational Approaches

Variational approaches are seen as one of the most promising direction in quantum machine learning [16]. They do not suffer from the input destruction problem, and have no limitations from the HHL algorithm or the failure of generalization of the quantum kernels.

Variational approaches iteratively update a parameterized quantum trial solution also called ansatz (from German ansatz = approach) using a classical optimization algorithm. The parameterized quantum trial solution is represented by a parametrized quantum circuit in the same way as a quantum kernel.

The variational approach can be used for binary classifier with two classes represented by the target values 0 and 1. With the input data vectors $x_{k}$ of dimension *m* and the binary output labels $t_{k}$ with a training set

$$D=\left\{\left(x_{1},t_{1}\right),\left(x_{2},t_{2}\right),\cdots ,\left(x_{N},t_{N}\right)\right\},\;\;t_{k}\in \left\{0,1\right\}.$$

A parametrized quantum circuit $U_{\phi (x_{k})}$ with *m* parameters encodes each input data vector $x_{k}$ of the dimension *m* [4]

(37) $$U_{\phi {\left(x\right)}_{k}}{|0\rangle }^{⊗m}.$$

Additionally, a variational quantum circuit represents the free parameter w

(38) $$U_{W(w)}$$

that will adapt during training by using an optimizer on a classical computer, leading to the state

(39) $$|\psi \left(x_{k},w\right)\rangle =U_{W(w)}\cdot U_{\phi {\left(x\right)}_{k}}{|0\rangle }^{⊗m}.$$

**Figure 3:**
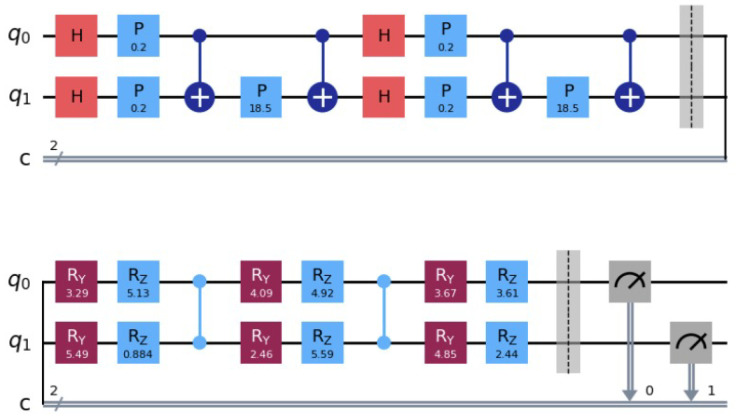
We use the parameterized circuit $U_{\phi (x)}=ZZFeatureMap$ with repetition for the input of dimension two mapping it into $2^{2}$ dimensional space. For $U_{W(w)}$ we use the TwoLocal circuit. It is a parameterized circuit consisting of alternating rotation layers and entanglement layers. An input vector $x_{k}$ defines the circuit $U_{\phi (x_{k})}$. We determine the output function $o_{k}\in \left\{0,1\right\}$. We minimize the loss function by the SPSA optimizer on a classical computer resulting in new parameter w that defines the adopted variational quantum circuit $U_{W(w)}$. We repeat the process using the adapted variational circuit for the next raining pattern until the error represented by the loss function is minimal. (The dashed lines are visual indicators of the grouping of a circuit sections).

The measured state $|\psi (x_{k},w)\rangle$ by a basis state $|q_{m}\cdots q_{1}q_{0}\rangle$ representing a binary string, see Figure 3. The measured binary string represents a parity function. A Boolean function whose value is one if and only if the input vector has an odd number of ones represents a parity function. For each input data vector $x_{k}$ we determine the output function $o_{k}\in \left\{0,1\right\}$ and perform an adaptation of the parameters w of the variational quantum circuit $U_{W(w)}$ using an optimizer on a classical computer that minimizes the loss function between the predicted values represented by the parity function of the measured basis state and the target values. The optimizer approximates a stochastic gradient descent by the Simultaneous Perturbation Stochastic Approximation (SPSA). SPSA requires only two measurements of the loss function, regardless of the dimension of the optimization problem [17] to estimate the stochastic gradient.

The advantages of classical deep learning models cannot be met by variational algorithms, like the principle of hierarchical organization or the representation of big training sets or regularization. The idea of hierarchical structures is based on the decomposition of a hierarchy into simpler parts leading to a more efficient way of representing information. This principle appears in nature; for example, the structure of the matter itself is hierarchically organized by elementary particles, atomic nuclei, atoms, and molecules [18]. The deep learning approach learns the hierarchical structure from the training data, leading to a high-level abstractions in data by architectures composed of multiple nonlinear transformations using gradient descent through error back-propagation. The back-propagation algorithm leads to a hierarchy from low-level structures to high-level structures, as demonstrated by natural complexity [19,20,21,22]. Variational quantum circuits cannot propagate the error backward, since this operation would require the determination of the activity of each layer for the classical optimizer; this operation can only be performed by measurement, which would lead to collapse. By increasing the number of layers in deep learning, we can increase the number of parameters. By doing so, we can add enough degrees of freedom to model large training sets. This is extremely helpful since nowadays, for specific tasks, a really large amount is collected. Ideally, to overcome overfitting, one has to use a model that has the right capacity. However, this task is difficult and costly since it involves searching through many different architectures. Many experiments with different number of neurons and hidden layers have to be conducted. Using an over-parameterized deep learning network, one can constrain it to the right complexity through regularization. The search for the correct model complexity can be conducted efficiently through empirical experiments. These advantages leading to the success of deep learning cannot be meet by variational algorithms.

## 6. Conclusions

The input destruction problem has not yet been solved, and theoretical speed-ups are usually analyzed by ignoring the input destruction problem. Other constraints are the normalized representation of vectors by amplitudes and quantum kernels with periodic receptive fields. We arrive at a dilemma: should we ignore or marginalize those constraints or not? Until now, no theoretical advantage over classical algorithms on real data was shown. It is questionable if the input destruction problem will be ever solved and if a qRAM is possible. Usually the constraints are ignored or marginalized.

Instead, we should determine if quantum machine learning (without the discussed constraints) can solve any problems more efficiently than a classical computer. Until now, such problems do not exist and we are far away from showing how to use quantum machine learning algorithms for real-world data. Are there any problems for which a quantum computer is more useful than a classical computer? If we do not answer these questions and overestimate the power of quantum machine, a quantum machine learning winter will follow in analogy to the AI winter.

What could be a possible answer? We can only speculate that in the future real quantum data will be generated through quantum physical experiments or that some new physical breakthroughs will lead to new efficient methods for the state preparation. Putting the speculation aside, quantum symbolic AI algorithms offer a better alternative for successful applications. This is because they avoid the input problem, they do not represent large training data by quantum states. Real-world applications, like the simulation of chemical reactions and optimization problems, should be investigated. The simulation of chemical reactions is based on Hamiltonian simulation together with variational quantum Eigensolvers [23]. Variational quantum Eigensolvers are based on a variational algorithm that estimates the ground state of a system [4,23]. Additionally, a promising direction is indicated by the Boltzmann machine [24,25,26] and deep belief networks [27,28] rather than the back-propagation algorithm.

## Data Availability

The original contributions presented in the study are included in the article, further inquiries can be directed to the corresponding author.
